# A comprehensive synthesis of dung beetle records (Coleoptera, Scarabaeidae, Scarabaeinae) from Sabah, Malaysia

**DOI:** 10.3897/BDJ.12.e126697

**Published:** 2024-09-12

**Authors:** Marx W-H. Yim, Xin Rui Ong, Li Yuen Chiew, Eleanor M. Slade

**Affiliations:** 1 Tropical Ecology & Entomology Lab, Asian School of the Environment, Nanyang Technological University, Singapore, Singapore Tropical Ecology & Entomology Lab, Asian School of the Environment, Nanyang Technological University Singapore Singapore

**Keywords:** Dung beetles, Coleoptera, Scarabaeidae, Scarabaeinae, Sabah, Malaysia, Borneo, GBIF

## Abstract

**Background:**

Dung beetles play key roles in terrestrial ecosystems, contributing to many important ecosystem process and functions, such as nutrient recycling, parasite control and seed dispersal. Due to their tight associations with mammals and their responses to environmental change, they are also frequently used as environmental and biological indicators. Despite their importance, knowledge about dung beetles in Southeast Asia is limited. To address this information gap, we established a databasing project - “Mobilising data on ecologically important insects in Malaysia and Singapore” - funded by the Global Biodiversity Information Facility (GBIF). As part of this project, we compiled two extensive datasets – a sampling-event and occurrence dataset and a taxonomic checklist – for the dung beetles of Sabah, Bornean Malaysia. The sampling-event dataset documents 2,627 unique sampling events and 21,348 dung beetle occurrence records for Sabah. The taxonomic checklist includes 156 confirmed dung beetle species and 36 synonyms, totalling 192 records. These datasets have been made open access through the GBIF portal, which we hope will enhance the understanding of dung beetle taxonomy and their distributions in Southeast Asia.

**New information:**

All data presented in this paper comprises of available information pertaining to the dung beetles of Sabah.

## Introduction

**Figure 1.**
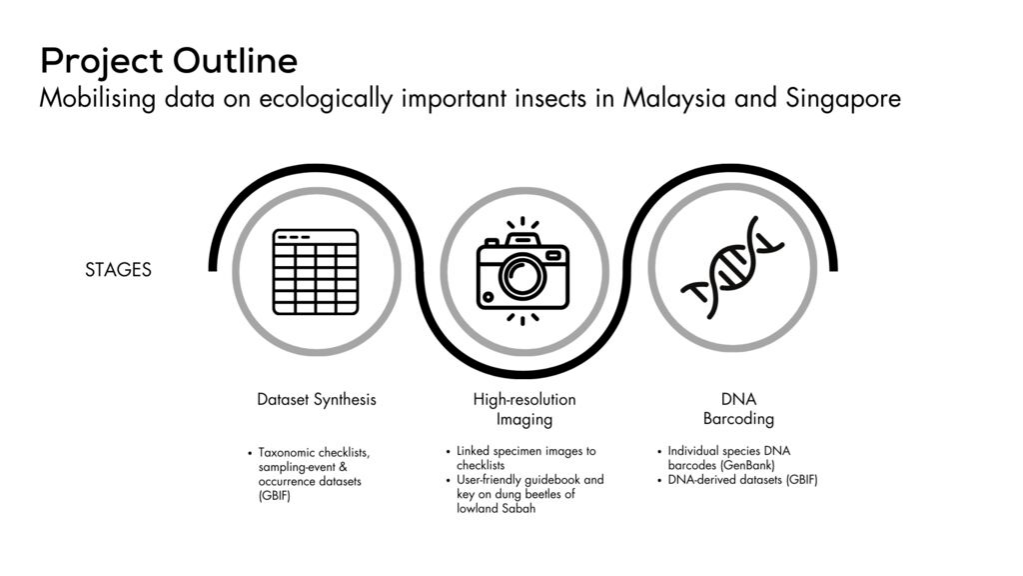
Project outline consisting of three stages and its deliverables of the project “Mobilising data on ecologically important insects in Malaysia and Singapore”.

Insects comprise around 80% of terrestrial animal diversity (Stork 2018). However, the distribution and taxonomy of many insect groups remains poorly documented, particularly in tropical ecosystems (Slade and Ong 2023). Dung beetles (Coleoptera, Scarabaeidae, Scarabaeinae) are a notable example. These beetles play crucial roles in ecosystem functioning, including soil enhancement, seed dispersal, parasite control and the reduction of greenhouse gases (Nichols et al. 2008, Beynon et al. 2012, Slade et al. 2016, Keller et al. 2021). Their close association with mammal communities, relying on their dung for sustenance and breeding and their sensitivity to environmental change underscores their importance in maintaining ecosystem functioning and their use as bioindicators (Davis et al. 2001, Nichols and Gardner 2011, Audino et al. 2014, Raine and Slade 2019, Fuzessy et al. 2021). Despite their ecological significance, a comprehensive understanding of the taxonomy and distribution of dung beetles is lacking for many regions and particularly in Southeast Asia. To address this data and knowledge gap, we developed two extensive datasets - a taxonomic checklist and a sampling-event and occurrence dataset for the dung beetles of Sabah, Malaysia. This forms part of a larger on-going project to mobilise data on dung beetles of Southeast Asia (Fig. 1).

We collated data from various sources, including taxonomic and ecological publications and published datasets. These data were prepared according to the Darwin Core Standard (DwC) and published open access through the Global Biodiversity Information Facility (GBIF) through a Biodiversity Information Fund for Asia (BIFA) funded project “Mobilising data on ecologically important insects in Malaysia and Singapore”. This project is on-going and is focused on mobilising data on dung beetles for Malaysia and Singapore. The project has multiple stages, including dataset synthesis, as well as high-resolution imaging and DNA barcoding of specimens (Fig. 1). In this paper, we focus on the development of the sampling-event and occurrence dataset and the taxonomic checklist. Our sampling-event dataset provides a comprehensive record of 2,627 unique sampling events from 1912 to 2022, encompassing over 21,348 dung beetle occurrence records across Sabah. The taxonomic checklist includes 192 records, comprising of 156 taxonomically accepted species names and 36 synonyms. In this paper, we describe the compilation of these datasets and provide a preliminary overview of the dung beetle species that occur in Sabah and their known distributions, including systematic sampling information and metadata. These datasets offer a comprehensive compilation of all known available data for dung beetles in Sabah, but have some limitations. The existing information has gaps in coverage, particularly at higher elevations and in less accessible regions. Species complexes and undescribed morpho-species are common in the original published datasets, but were omitted according to GBIF guidelines. We therefore caution that extra attention should be given to the spatial, temporal and taxonomic resolution when conducting any analysis using these datasets.

These datasets play a vital role in enhancing our understanding of dung beetle taxonomy and distribution within Southeast Asia, addressing broader challenges in tropical insect conservation (Slade and Ong 2023). Datasets such as these are essential for long-term monitoring efforts, particularly in evaluating how species respond to land-use and climate change over time. An important aspect of the overall project is in promoting an integrative taxonomic approach which combines molecular delimitation and morphological information for species identification. Specimens obtained through this project will be sequenced to generate individual DNA barcodes which will be published as a molecular barcode dataset and linked to the datasets published here. High-resolution images of each species will also be linked to these datasets alongside the creation of a comprehensive guide and key to the dung beetles of lowland Sabah.

## General description

### Purpose

The project “Mobilising data on ecologically important insects in Malaysia and Singapore” seeks to address the existing gaps in dung beetle taxonomy and distribution while also increasing the representation of Southeast Asian dung beetles in GBIF-mediated data. The primary objective of the project is to mobilise and digitise georeferenced records, creating open-access GBIF datasets with centralised and standardised data. These datasets are structured according to GBIF guidelines, including sampling-event and occurrence datasets and taxonomic checklists. The process of creating these datasets is covered in this data paper. In addition to providing high-quality datasets, the broader goals of the project include high-resolution imaging of specimens to create user-friendly guides and keys and DNA barcoding of specimens to help deconflict morphospecies and manuscript names and help resolve the complex taxonomy of dung beetles in the region. Through this integrative taxonomic approach, we hope to begin to overcome some of the the existing taxonomic and capacity impediments present in entomology in the region, to promote the use of dung beetles as bioindicators and to encourage their use in ecological monitoring and research projects.

## Project description

### Title

BIFA6_032 Mobilising data on ecologically important insects in Malaysia and Singapore

### Personnel

- **Project PI**: Eleanor Slade (Dr., Associate Professor, Tropical Ecology & Entomology Lab, Asian School of the Environment, Nanyang Technological University);
- **Core Team**: Marx Yim (Project Officer, Tropical Ecology & Entomology Lab, Asian School of the Environment, Nanyang Technological University), Ong Xin Rui (PhD Student, Tropical Ecology & Entomology Lab, Asian School of the Environment, Nanyang Technological University), Chiew Li Yuen (Dr., Postdoctoral Researcher, Tropical Ecology & Entomology Lab, Asian School of the Environment, Nanyang Technological University);
- **Collaborators**: Arthur Chung (Forest Research Centre, Sabah, Malaysia), Ang Yu Chen (Lee Kong Chian Natural History Museum, Singapore), Maria Heikkila (Finnish Museum of Natural History, Luomos, Finland), Heidi Viljanen (Finnish Museum of Natural History, Luomos, Finland) and Monica Suleiman (Borneensis, Universiti Malaysia Sabah, Malaysia).

### Study area description

Sabah and Sarawak, Bornean Malaysia and Singapore. In this paper, we cover datasets for Sabah, Malaysia only.

### Design description

This project involves: (i) dataset synthesis, (ii) high-resolution imaging and (iii) DNA barcoding of specimens. See Fig. 1 for project outline.

(i) Dataset synthesis

**Figure 2.**
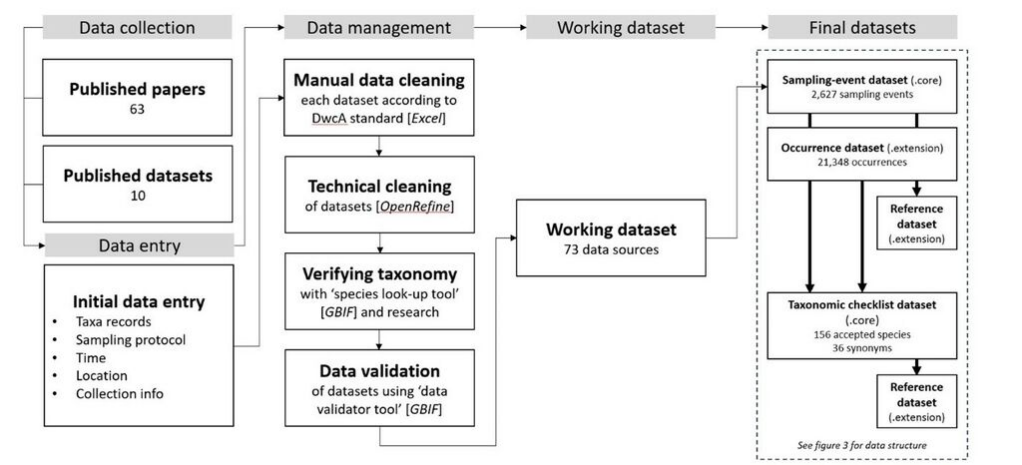
The workflow for the dataset synthesis comprises multiple steps. Verbatim data derived from all available data sources related to Sabah dung beetles were initially compiled into an Excel template. After manual and technical cleaning, including taxonomic verification and validation, a refined working dataset was created. This working dataset was then partitioned into the final datasets for publication.

During the *Data Collection* step, we aggregated all relevant sources, including published scientific literature and datasets, while also identifying museums and repositories housing essential dung-beetle collections. In the subsequent *Data Entry* step, information was extracted verbatim from the sources and input into preliminary datasets. Following this, in the *Data Management* step, all verbatim data underwent standardisation according to the Darwin Core Archive (DwC-A) biodiversity informatics standard. This step also encompassed technical cleaning to rectify errors using OpenRefine, taxonomy verification utilising the GBIF Species Checker Tool and data validation with the GBIF Validator tool. For a detailed overview of the workflow, please refer to Fig. 2.

(ii) High resolution imaging of specimens

As an ongoing part of the project, key specimens are being curated and imaged in the Image Capturing stage. These images will play a vital role in species identification, resolving conflicts in manuscript names and morphospecies. These images will also contribute to the ongoing development of a key and guide to the lowland dung beetles of Sabah. In a subsequent phase, the images will be linked to each species record within the taxonomic checklist.

(iii) DNA barcoding of specimens

To further validate and confirm the identity of a species and to help resolve species complexes and morphospecies identifications, specimens will be sequenced in the DNA barcoding stage using Next-Generation Sequencing. This will be published as a molecular barcode extension dataset at a later time.

## Sampling methods

### Study extent

Sabah, Bornean Malaysia

### Sampling description

The dataset synthesis comprises of multiple steps. See Fig. 2 for the complete workflow of the data synthesis.

### Step description

**Data collection**

The biodiversity data presented in the sampling-event and occurrence dataset and taxonomic checklist dataset were derived from 63 published papers and from 10 published datasets (Fig. 2) which focused on dung beetles in Sabah, Malaysia. References for all 73 data sources are available in the bibliographicCitation column within the sampling-event core and are also accessible in the reference extension linked to the core.

**Data entry**

We compiled all relevant verbatim data related to taxonomy, occurrence and sampling protocol information and all other relevant metadata into a single, comprehensive dataset held at the Tropical Ecology & Entomology Lab as the primary repository for all initial raw data entry. All data were manually entered into a common Microsoft Excel template. The following minimum set of variables were collected: scientific name, species counts, collection date, locality, geographic coordinates (i.e. latitude, longitude) and sampling protocol information (i.e. trap type, bait type, transect distance, trap spacing). No transformation of verbatim data occurred at this point.

**Data management**

The original dataset containing verbatim data was subsequently converted to adhere to the Darwin Core Archive (DwC-A) biodiversity informatics data standards. These datasets underwent a process of data cleaning to rectify typographical errors, ensure consistency in vocabulary and identify outliers. This process was conducted using OpenRefine v.3.3 (https://openrefine.org) and R. The data were then subjected to validation using the GBIF 'data validator' tool (https://www.gbif.org/tools/data-validator) and the taxonomy of an interim species checklist was confirmed using the GBIF 'species look-up' tool (https://www.gbif.org/tools/species-lookup). Following these steps, a finalised working dataset was prepared for segmentation into respective final datasets intended for publication on the Biodiversity Information Fund for Asia (BIFA) Integrated Publishing Toolkit (IPT) hosted by GBIF.

**Final datasets**

**Figure 3.**
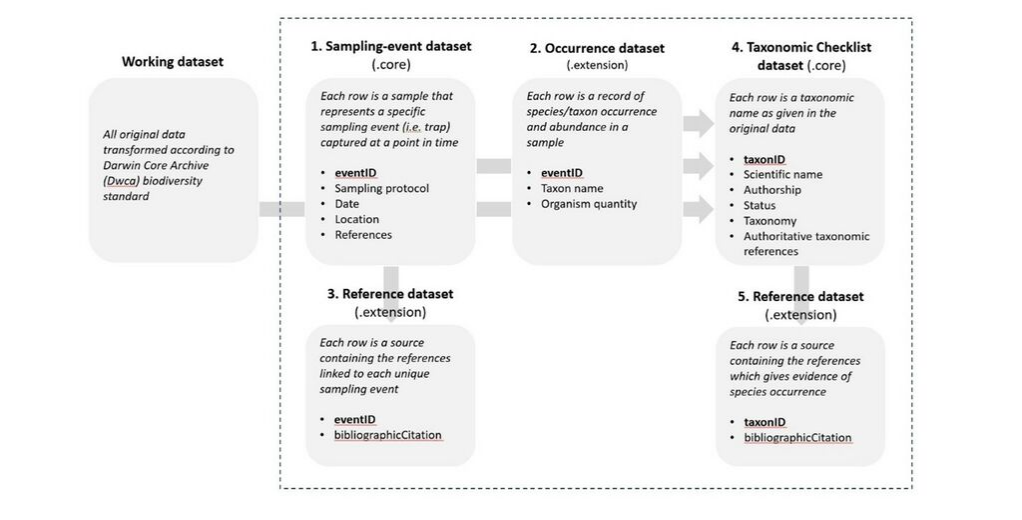
Data structure of each dataset (core and extension) and their key variables. Unique identifiers which link the core and extension datasets are in bold within the grey boxes.

The sampling-event core, serving as the foundation for the final datasets (see Table 1), encompasses 2,627 unique sampling-events. Linked to this core is the occurrence extension, which contains 21,348 individual occurrences. The taxonomic checklist, which includes 192 dung beetle records consisting of 156 accepted species names and 36 synonyms (see Table 2), is derived from the occurrence dataset extension. A reference extension listing all 73 data sources is provided as an extension of the sampling-event core. Please refer to Table 1 for a summary of the final datasets. Each dataset adheres to specific data quality requirements and recommendations established by GBIF (see Fig. 3) to ensure the quality, completeness and overall value of the datasets. The dataset cores are interconnected with extensions using unique identifiers (Table 1, Fig. 3). All column names are controlled vocabulary as set out by DwC-A (see Table 3). Prior to publication on the GBIF IPT, all final datasets underwent secondary data cleaning using OpenRefine and validation using the GBIF ‘data validator’ tool. The taxonomic checklist was re-verified using the GBIF ‘species look-up’ tool.

- Sampling-event and Occurrence Records of Dung Beetles (Coleoptera, Scarabaeidae, Scarabaeinae) from Sabah, Malaysia Borneo (Yim et al. 2024b)

Serving as a dataset core, the sampling-event dataset comprises of rows representing unique sampling events and is represented by a unique identifier (eventID). Each sampling event contains a column, samplingProtocol, which contains protocol information such as trap type, dung type, no. of transects, no. of traps, transect length and between-trap distance. Notably, the eventRemark column is important as it determines the scale of data (i.e. trap-, site-, study-level data or taxonomic-). Most derived occurrences were sourced from data sources offering trap-level data. However, some were derived from sources that aggregated their data, presenting only site- and study-level information. Some taxonomic papers lacked individual abundance data, resulting in only species level rather than individual level occurrence data. Emphasis is placed on examining this column, as the scale the data were recorded at will determine the subsequent analysis that can be performed. This column also contains other relevant sampling protocol information, such as verbatim site name, transect name/no. and trap no. Cells containing more than one type of data are separated by ‘|’.

All rows have an eventDate which is standardised according to yyyy-mm-dd to demarcate a specific date (see Table 3 for other ways an eventDate can be arranged). Geographic coordinates are provided in decimal degrees for latitude and longitude and checked using Google Maps. In instances where verbatim geographic coordinates are absent from the source, coordinates were estimated based on the locality and other available information. Each unique event can be linked back to its source through the column bibliographicCitation. A reference extension is connected to the sampling-event core using the unique identifier, eventID. Each row in the reference extension represents the bibliographic citation of each unique event.

The sampling-event dataset is supplemented by an occurrence extension which comprises the associated occurrences of all the unique sampling events found in the core. In this extension, each row is a record that was gathered during the event. Each record contains a single species name followed by the quantity that was collected. Morphospecies data were excluded in accordance with GBIF guidelines. Each occurrence can be linked back to the sampling-effort dataset through the unique identifier (eventID). The parentEventID serves to identify event(s) that occurred together, which means it is from a same single study (see Fig. 2). Detailed descriptions of fields/variables/controlled vocabulary can be found in Table 3.

- Taxonomic Checklist of the Dung Beetles (Coleoptera, Scarabaeidae, Scarabaeinae) of Sabah, Malaysia (Yim et al. 2024a)

Within the core of the taxonomic checklist, each row represents either an accepted species name or a synonym. The scientific names are split into higher taxonomic classifications (Kingdom, Phylum, Class, Order, Family, Subfamily, Genus, Subgenus, infragenericEpithet, specificEpithet) with the assistance of the ‘GBIF species-lookup tool’ and individually verified. Any missing information, such as subgenus, were obtained from the latest available taxonomic literature. Species names not recognised by GBIF, but that have been taxonomically accepted, were input manually. See Table 2 for the full list of accepted species and synonyms.

Authorships were authenticated, with a specific focus on the application of parentheses (round brackets). In instances where the species was described under a genus different from the original description, parentheses were used around the author and year, indicating the existence of a synonym. This is reflected in the taxonomic status column (i.e. accepted, synonym). After determining taxon rank and taxonomic status, the accepted names and original names of each species were confirmed. In cases where a record was a synonym, the accepted name of the species, if not already present, was added into the checklist, ensuring the taxonomic checklist comprises all accepted names of dung beetle species in Sabah. Basionyms of accepted species names were included into the checklist only if they originated from the utilised sources. Morphospecies from the original data were not included as per GBIF guidelines. Synonyms and accepted names were linked with unique identifiers (taxonID) using the acceptedNameUsageID and originalnameUsageID columns. Two taxon references were provided in the columns, namePublishedin and nameAccordingTo. The former contains the reference in which the species name was first established, while the later reference serves as the authoritative taxonomic reference for the record.

The taxonomic checklist is supplemented by a reference extension, with both linked through the unique identifier, taxonID. Each row in the reference extension corresponds to the bibliographic citation of the taxon occurrence.

## Geographic coverage

### Description

**Figure 4.**
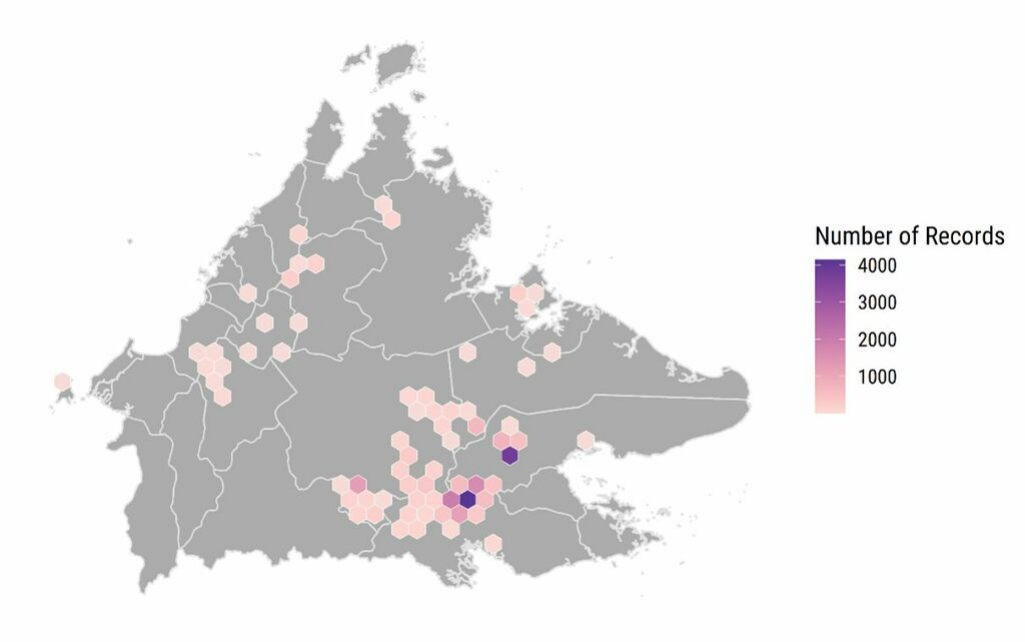
Distribution of occurrence records represented in the datasets, with the number of records represented in hexagonal grids of approximately 100 km^2^.

This data paper encompasses all available data of known dung beetle records in Sabah of Borneon Malaysia. See Fig. 4 for distribution of occurrence records represented in the datasets.

### Coordinates

3.908 and 7.406 Latitude; 113.928 and 119.377 Longitude.

## Taxonomic coverage

### Description

**Figure 5.**
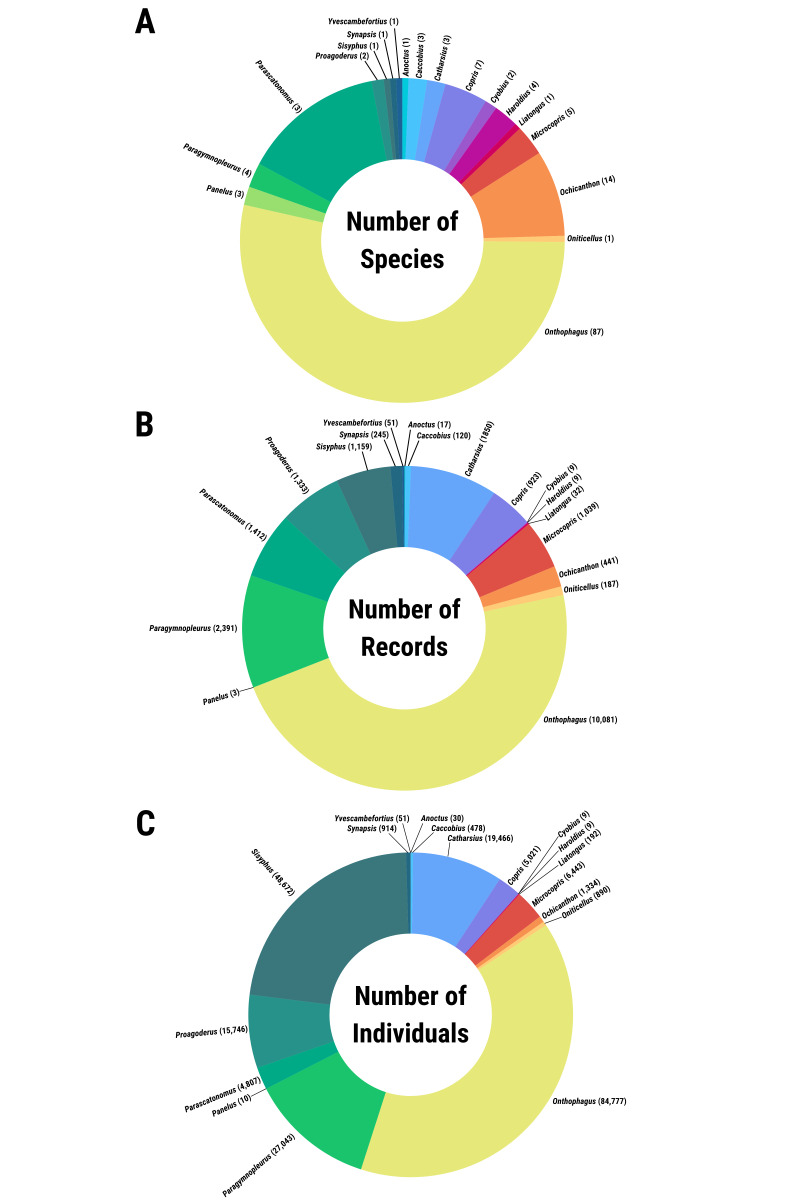
Pie charts showing the number of species (A), occurrence records (B) and recorded individuals per genus in the datasets (C).

These datasets consist of all known dung beetle occurrence records taxonomically described from Sabah, Malaysia. In total, 20 genera, 156 accepted species and 36 synonyms are represented (Table 2). This summary is derived from 21,348 occurrences constituting 217,270 individual dung beetles (see Fig. 5). It is important to note that the coverage is not exhaustive as the map shows that there are locations yet to be surveyed for dung beetles, particularly at higher elevations and in more remote regions. Nevertheless, this represents the most comprehensive effort to compile a unified taxonomic checklist and sampling-effort and occurrence dataset for the dung beetles of Sabah. There were also many morphospecies within the datasets examined that are not included within this dataset as they are not described species. The next steps are to resolve and describe new or cryptic species and to undertake genetic sequencing to create individual DNA barcodes that can be linked to GenBank (https://www.ncbi.nlm.nih.gov/genbank/). These DNA barcoding data will then be published alongside these existing datasets.

### Taxa included

**Table:** 

Rank	Scientific Name	
kingdom	Animalia	
phylum	Arthropoda	
subphylum	Arthropoda	
class	Insecta	
order	Coleoptera	
family	Scarabaeidae	
subfamily	Scarabaeinae	Dung beetles
genus	*Anoctus*	
genus	*Caccobius*	
genus	*Catharsius*	
genus	*Copris*	
genus	*Cyobius*	
genus	*Gymnopleurus*	
genus	*Haroldius*	
genus	*Liatongus*	
genus	*Microcopris*	
genus	*Ochicanthon*	
genus	*Oniticellus*	
genus	*Onthophagus*	
genus	*Panelus*	
genus	*Paragymnopleurus*	
genus	*Parascatonomus*	
genus	*Phacosoma*	
genus	*Proagoderus*	
genus	*Sisyphus*	
genus	*Synapsis*	
genus	*Yvescambefortius*	

## Temporal coverage

### Notes

From 1912 to 2022

## Usage licence

### Usage licence

Creative Commons Public Domain Waiver (CC-Zero)

### IP rights notes

These datasets on the Sabah dung beetles are fully open-access and other researchers are encouraged to share and adapt the data for their own research. When doing this researchers are encouraged to: (i) give appropriate attribution to the data providers and cite both the dataset and its accompanying publication, in accordance with the Creative Commons Attribution Non-Commercial CC-BY-NC 4.0 License, (ii) take note of the representativeness and temporal and spatial resolution of the data, (iii) feedback any issues you face, (iv) get in touch with us at Eleanor Slade (eleanor.slade@ntu.edu.sg) or Marx Yim (marx.yim@ntu.edu.sg) if you have any questions. Each dataset core will have dataset extensions and data may need to be combined and summarised for further analysis by linking the sheets through the IDs (i.e. eventID, taxonID). For analysis of events and occurrences, please always refer to “eventRemarks” to determine data type and data scale (i.e. trap-, site-, study-, taxonomic-). For species-level data, please refer to “taxonomicStatus” to avoid confusion between an accepted species and a synonym. It is essential to note that morphospecies data have been excluded from the datasets in accordance with GBIF guidelines.

These datasets represent our ongoing efforts to advance the knowledge of dung beetles in Sabah and the broader region. They will be curated and expanded as new data become available. Each update and inclusion of fresh data will result in the creation of a new version, so researchers should be aware of the specific version they are working with. In addition to these datasets, we are actively working on two extensions: a molecular barcode extension and a digital image extension. These extensions will be made openly available soon.

## Data resources

### Data package title

A comprehensive synthesis of dung beetle records (Coleoptera, Scarabaeidae, Scarabaeinae) from Sabah, Malaysia

### Number of data sets

2

### Data set 1.

#### Data set name

Sampling event and occurrence records of dung beetles (Coleoptera, Scarabaeidae, Scarabaeinae) from Sabah, Malaysia Borneo

#### Data format

Darwin Core Archive (DwC-A)

#### Download URL

https://www.gbif.org/dataset/822991fc-0532-46b0-8dda-d17dd6cdb8df

#### Description

This sampling event and occurrence dataset comprises 2,627 unique sampling events, documenting a total of 21,348 individual occurrences of dung beetles in Sabah, Malaysian Borneo. The data is derived from 63 published papers and from 10 published datasets.

**Data set 1.** 

Column label	Column description
parentEventID	An identifier for the broader event that groups this and other events. This is a globally unique identifier.
eventID	An identifier for the set of information associated with an event (something that occurs at a place and time). It is used as a unique identifier of each event and can be interlinked between the available extensions and the core dataset. This is a globally unique identifier.
samplingProtocol	The names of, references to, or descriptions of the methods or protocols used during an event.
eventRemarks	Comments or notes about the event. Emphasis is placed on examining this column, as the data type (i.e. trap-, site-, study-, taxonomic-) will determine the subsequent analysis that can be performed. This column also contains other sampling such as verbatim site name, transect name/no. and trap no. Cells containing more than one type of data are separated by ‘|’.
samplingEffort	The amount of effort expended during an event.
sampleSizeValue	A numeric value for a measurement of the size (time, duration, length, area or volume) or a sample in a sampling event.
sampleSizeUnit	The unit of measurement of the size (time, duration, length, area or volume) of a sample in a sampling event.
eventDate	The date-time or interval during which an event occurred. Examples:· 2018-08-29 (some time during 29 August 2018)· 1906-06 (some time in June 1906)· 1971 (some time in 1971)· 2007-03-01/2008-05-11 (some time during the interval between 1 March 2007 and 11 May 2008)· 1900/1909 (some time during the interval between the beginning of the year 1900 and the end of the year 1909)· 2007-11-13/15 (some time in the interval between 13 November 2007 and 15 November 2006).
country	Country in which event occurred.
countryCode	The standard code for the country in which the event occurred. Country code is as per ISO 3166-1-alpha-2 country code.
locality	The specific description of the place.
decimalLatitude	The geographic latitude (in decimal degrees, using the spatial reference system given in geodeticDatum) of the geographic centre of the event.
decimalLongitude	The geographic longitude (in decimal degrees, using the spatial reference system given in geodeticDatum) of the geographic centre of the event.
geodeticDatum	The ellipsoid, geodetic datum or spatial reference system (SRS), upon which the geographic coordinates given in decimalLatitude and decimalLongitude are based.
associatedReferences	A reference of where the event was derived.
basisofRecord	The specific nature of the data record (i.e. MaterialCitation).
scientificName	The scientific name of the species, with full authorship and date information if known.
organismQuantity	The number or value for the quantity or organism gathered from the event.
organismQuantityType	The type of quantification system used for the quantity of the organisms.
occurrenceStatus	A statement about the presence or absence of a taxon at a location.
taxonRank	The taxonomic rank of the most specific name in the scientificName.
type	The nature or genre of the resource (i.e. Event).
kingdom	The full scientific name of the kingdom in which the Taxon is classified.
phylum	The full scientific name of the phylum in which the Taxon is classified.
class	The full scientific name of the class in which the Taxon is classified.
order	The full scientific name of the order in which the Taxon is classified.
family	The full scientific name of the family in which the Taxon is classified.

### Data set 2.

#### Data set name

Taxonomic checklist of the dung beetles (Coleoptera, Scarabaeidae, Scarabaeinae) of Sabah, Malaysia Borneo

#### Data format

Darwin Core Archive (DwC-A)

#### Download URL

https://www.gbif.org/dataset/c41029b7-b950-48f3-be69-60267043e020

#### Description

This taxonomic checklist dataset presents 192 dung beetle records consisting of 156 accepted species names and 36 synonyms of the dung beetles of Sabah, Malaysia. These data are derived from the occurrence records from 63 published papers and 10 published datasets.

**Data set 2.** 

Column label	Column description
taxonID	A unique identifier for the taxon and can be used to linked to available extensions of the core dataset. This is a globally unique identifier.
kingdom	The full scientific name of the kingdom in which the Taxon is classified.
phylum	The full scientific name of the phylum in which the Taxon is classified.
class	The full scientific name of the class in which the Taxon is classified.
order	The full scientific name of the order in which the Taxon is classified.
family	The full scientific name of the family in which the Taxon is classified.
subfamily	The full scientific name of the subfamily in which the Taxon is classified.
genus	The full scientific name of the genus in which the Taxon is classified.
subgenus	The full scientific name of the subgenus in which the Taxon is classified.
infragenericEpithet	The infrageneric part of a binomial name at ranks above species, but below genus.
specificEpithet	The name of the first or species epithet of the scientificName.
scientificName	The scientific name of the species, with full authorship and date information, if known.
scientificNameAuthorship	The latest known author of the scientific name.
nameAccordingTo	The reference to the source in which the specific taxon is defined. This is typically the latest authoritative taxonomic reference to species.
namePublishedIn	A reference for the publication in which the scientificName was originally established.
namePublishedInYear	The year in which the scientificName was published.
taxonomicStatus	The taxonomic status of the scientificName as determined by expert opinion.
acceptedNameUsage	The full name, with authorship and date information if known, of the currently valid or accepted Taxon.
acceptedNameUsageID	An identifier of the name usage of the current valid or accepted taxon. This is a globally unique identifier.
originalNameUsage	The taxon name, with authorship and date information, if known, as it originally appeared when first established. This is typically the basionym of the scientificName or senior/earlier homonym for replaced names.
originalNameUsageID	An identifier for the name usage in which the scientific name was originally established. This is a globally unique identifier.
taxonRank	The taxonomic rank of the most specific name in the scientificName.
taxonRemarks	Comments of notes about the taxon or name.
bibliographicCitation	A bibliographic reference of the resource.

## Figures and Tables

**Table 1.** Summary of final datasets detailing the dataset type, subtype and unique identifier linking the core dataset to an extension dataset.

#	**Dataset name**	**Dataset type**	**Dataset subtype**	**Unique identifier**
1	Sampling Event and Occurrence Records of Dung Beetles (Coleoptera, Scarabaeidae, Scarabaeinae) from Sabah, Malaysia	Sampling-Event	Core	eventID
2	Occurrence dataset	Occurrence	Extension	eventID
3	Reference	Reference	Extension	eventID
4	Taxonomic Checklist of the Dung Beetles (Coleoptera, Scarabaeidae, Scarabaeinae) of Sabah, Malaysia	Checklist	Core	taxonID
5	Reference	Reference	Extension	taxonID

**Table 2.** An overview of the species names within the taxonomic checklist dataset.

**Scientific Name**	**Taxonomic Status**	**Taxon Rank**
*Anoctuslaevis* Sharp, 1875	accepted	species
*Caccobiusbinodulus* Harold, 1877	accepted	species
*Caccobiusunicornis* (Fabricius, 1798)	accepted	species
*Caccobiusbawangensis* Ochi, Kon & Kikuta, 1997	accepted	species
*Catharsiusmolossus* (Linnaeus, 1758)	proParteSynonym	species
*Catharsiusdayacus* Lansberge, 1886	accepted	species
*Catharsiusrenaudpauliani* Ochi & Kon, 1996	accepted	species
*Coprisagnus* Sharp, 1875	accepted	species
*Coprisgibbulus* Lansberge, 1886	accepted	species
*Coprisgibbulusborneensis* Ochi & Kon, 2005	accepted	subspecies
*Coprisnuma* Lansberge, 1886	accepted	species
*Coprispoggii* Ochi & Kon, 2005	accepted	species
*Coprisreflexus* Panzer, 1794	synonym	species
*Coprisramosiceps* Gillet, 1921	accepted	species
*Coprissinicus* Hope, 1842	accepted	species
*Cyobiuscheyi* Ochi, Kon & Kashizaki, 2006	accepted	species
*Cyobiuswallacei* Sharp, 1875	accepted	species
*Gymnopleurusmaurus* Sharp, 1875	synonym	species
*Gymnopleurussparsus* Sharp, 1875	synonym	species
*Haroldiusborneensis* Paulian, 1993	accepted	species
*Haroldiusdiscoidalis* Paulian, 1993	accepted	species
*Haroldiuspauliani* Scheuern, 1995	accepted	species
*Haroldiusrugatulus* Boucomont, 1914	accepted	species
*Liatongusfemoratus* (Illiger, 1800)	accepted	species
*Microcoprisdoriae* (Harold, 1877)	accepted	species
*Microcoprisfujiokaiporingensis* Ochi & Kon, 2005	accepted	subspecies
*Microcoprishidakai* Ochi & Kon, 1996	accepted	species
*Microcoprisreflexus* (Fabricius, 1787)	accepted	species
*Ochicanthoncrockermontis* Krikken & Huijbregts, 2007	accepted	species
*Ochicanthondanum* Krikken & Huijbregts, 2007	accepted	species
*Ochicanthondytiscoides* (Boucomont, 1914)	accepted	species
*Ochicanthongangkui* (Ochi, Kon & Kikuta, 1997)	accepted	species
*Ochicanthonhikidai* (Ochi, Kon & Kikuta, 1997)	accepted	species
*Ochicanthonkikutai* Ochi, Ueda & Kon, 2006	accepted	species
*Ochicanthonkimanis* Krikken & Huijbregts, 2007	accepted	species
*Ochicanthonmaryatiae* Ochi, Ueda & Kon, 2006	accepted	species
*Ochicanthonmasumotoi* (Ochi & Araya, 1996)	accepted	species
*Ochicanthonparantisae* (Ochi, Kon & Kikuta, 1997)	accepted	species
*Ochicanthonrombauti* Krikken & Huijbregts, 2007	accepted	species
*Ochicanthontambunan* Krikken & Huijbregts, 2007	accepted	species
*Ochicanthonworoae* Ochi, Ueda & Kon, 2006	accepted	species
*Oniticellussarawacus* Gillet, 1926	synonym	species
*Oniticellustessellatus* Harold, 1879	accepted	species
*Onthophagusfujiii* Ochi & Kon, 1995	accepted	species
*Onthophaguslimbatus* (Herbst, 1789)	accepted	species
*Onthophagusluridipennis* Boheman, 1858	accepted	species
*Onthophagusnigriobscurior* Ochi, Kon & Tsubaki, 2009	accepted	species
*Onthophagusparviobscurior* Ochi, Kon & Tsubaki, 2009	accepted	species
*Onthophaguscheyi* Ochi & Kon, 2006	accepted	species
*Onthophagusdanumensis* Ochi, Kon & Barclay, 2009	accepted	species
*Onthophagushikidai* Ochi & Kon, 2006	accepted	species
*Onthophagusliwagensis* Ochi & Kon, 2006	accepted	species
*Onthophagusmasaoi* Ochi, 1992	accepted	species
*Onthophagusparamasaoi* Ochi, Kon & Barclay, 2009	accepted	species
*Onthophagusworoae* Ochi & Kon, 2006	accepted	species
*Onthophagusyumotoi* Ochi & Kon, 2006	accepted	species
*Onthophagusdiabolicus* Harold, 1877	accepted	species
*Onthophagusarayai* Ochi & Kon, 2007	accepted	species
*Onthophagusdeliensis* Lansberge, 1885	accepted	species
*Onthophagustridentitibialis* Ochi & Kon, 2008	accepted	species
*Onthophagusangustatus* Boucomont, 1914	accepted	species
*Onthophagusaphodioides* Lansberge, 1883	accepted	species
*Onthophagusazusae* Ochi & Kon, 2006	accepted	species
*Onthophagusbatillifer* (Sharp, 1875)	accepted	species
*Onthophagusborneensis* Harold, 1877	accepted	species
*Onthophaguscervicapra* Boucomont, 1914	accepted	species
*Onthophaguscupreopastillatus* Ochi & Kon, 2006	accepted	species
*Onthophagusfalculatus* Boucomont, 1914	accepted	species
*Onthophagushosomai* Ochi & Kon, 2014	accepted	species
*Onthophagusincisus* Harold, 1877	accepted	species
*Onthophagusishiii* Ochi & Kon, 1995	accepted	species
*Onthophaguskashizakii* (Ochi & Kon, 2005)	accepted	species
*Onthophaguskawaharai* Ochi & Kon, 2007	accepted	species
*Onthophagusmagnioculus* Ochi & Kon, 2006	accepted	species
*Onthophagusmatsuii* Ochi & Kon, 2006	accepted	species
*Onthophagusmegapacificus* Ochi & Kon, 2006	accepted	species
*Onthophagusobscurior* Boucomont, 1914	accepted	species
*Onthophagusopacihartiniae* Ochi & Kon, 2015	accepted	species
*Onthophagusotai* Ochi & Kon, 2006	accepted	species
*Onthophaguspacificus* Lansberge, 1885	accepted	species
*Onthophaguspastillatus* Boucomont, 1919	accepted	species
*Onthophagusphillippsorum* Krikken & Huijbregts, 1987	accepted	species
*Onthophagusrobertopoggii* Ochi & Kon, 2006	accepted	species
*Onthophagusrutilans* Sharp, 1875	accepted	species
*Onthophagusrutilansaborneensis* Ochi, Kon & Tsubaki, 2009	accepted	subspecies
*Onthophagussabahensis* Ochi & Kon, 2006	accepted	species
*Onthophagussepilokensis* Ochi & Kon, 2006	accepted	species
*Onthophagussimboroni* Ochi & Kon, 2006	accepted	species
*Onthophagusvulpes* Harold, 1877	accepted	species
*Onthophaguswaterstradti* Boucomont, 1914	accepted	species
*Onthophagustrituber* (Wiedemann, 1823)	accepted	species
*Onthophagusanitidus* Ochi & Kon, 2005	synonym	species
*Onthophagusbrendelli* Ochi, Kon & Barclay, 2008	synonym	species
*Onthophagusbundutuhanensis* Ochi, Kon & Barclay, 2008	synonym	species
*Onthophagusdanumcupreus* Krikken & Huijbregts, 2009	synonym	species
*Onthophagusfujiokai* Ochi & Araya, 1996	synonym	species
*Onthophagusgunsalami* Ochi & Kon, 2005	synonym	species
*Onthophaguskatoi* Ochi & Araya, 1996	synonym	species
*Onthophaguskatoiporingensis* Ochi & Kon, 2005	synonym	subspecies
*Onthophaguskikutai* Ochi & Kon, 2005	synonym	species
*Onthophagusliewi* Ochi & Kon, 2005	synonym	species
*Onthophagusmonticupreus* Krikken & Huijbregts, 2009	synonym	species
*Onthophaguspenicillatus* Harold, 1879	synonym	species
*Onthophagusporingensis* Ochi & Kon, 2005	synonym	species
*Onthophagusrudis* Sharp, 1875	synonym	species
*Onthophagussarawacus* Harold, 1877	synonym	species
*Onthophagussayapensis* Ochi & Kon, 2005	synonym	species
*Onthophagussemiaureus* Lansberge, 1883	synonym	species
*Onthophagussemicupreus* (Harold, 1877)	synonym	species
*Onthophagustaichii* Ochi, Kon & Barclay, 2008	synonym	species
*Onthophagustamijii* Kon, Sakai & Ochi, 2000	synonym	species
*Onthophagushidakai* Ochi & Kon, 1995	accepted	species
*Onthophagusjohkii* Ochi & Kon, 1994	accepted	species
*Onthophagusschwaneri* Snellen Van Vollenhoven, 1864	synonym	species
*Onthophaguswatanabei* Ochi & Kon, 2002	synonym	species
*Onthophagusquasijohkii* Ochi & Kon, 2005	accepted	species
*Onthophagusborneotagal* Ochi, Kon & Barclay, 2016	accepted	species
*Onthophaguschandrai* Ochi, 2007	accepted	species
*Onthophagushiroyukii* Ochi, 2007	accepted	species
*Onthophaguskoni* Ochi, 2007	accepted	species
*Onthophagusmaryatiae* Ochi & Kon, 2005	accepted	species
*Onthophagusquasitagal* Ochi & Kon, 2005	accepted	species
*Onthophaguslaevis* Harold, 1880	accepted	species
*Onthophaguslaevislaevis* Harold, 1880	accepted	subspecies
*Onthophagusmulleri* Lansberge, 1883	accepted	species
*Onthophagussagittarius* (Fabricius, 1775)	accepted	species
*Onthophagussumatranus* Lansberge, 1883	accepted	species
*Onthophagusblumei* Lansberge, 1883	accepted	species
*Onthophagusaereopictus* Boucomont, 1914	accepted	species
*Onthophagusaurifex* Harold, 1877	synonym	species
*Onthophagusbangueyensis* Boucomont, 1914	accepted	species
*Onthophagusclivimerus* Huijbregts & Krikken, 2011	accepted	species
*Onthophagusdeflexicollis* Lansberge, 1883	accepted	species
*Onthophagusdux* Sharp, 1875	synonym	species
*Onthophagusfoedus* Boucomont, 1914	accepted	species
*Onthophagusjavaecola* Balthasar, 1959	accepted	species
*Onthophaguslilliputanus* Lansberge, 1883	accepted	species
*Onthophagusmentaveiensis* Boucomont, 1914	accepted	species
*Onthophagusochromerus* Harold, 1877	accepted	species
*Onthophaguspavidus* Harold, 1877	accepted	species
*Onthophagusphanaeides* Frey, 1956	accepted	species
*Onthophagusrorarius* Harold, 1877	accepted	species
*Onthophagusrouyeri* Boucomont, 1914	accepted	species
*Onthophagusrugicollis* Harold, 1880	accepted	species
*Onthophagussideki* Krikken & Huijbregts, 1987	accepted	species
*Onthophagussubcornutus* Boucomont, 1914	accepted	species
*Onthophagustaeniatus* Boucomont, 1914	accepted	species
*Onthophagusvethi* Krikken, 1977	accepted	species
*Onthophagushirsutulus* Lansberge, 1883	accepted	species
*Onthophaguspeninsularis* Boucomont, 1914	accepted	species
*Panelusdanumensis* Ochi, Kon & Barclay, 2009	accepted	species
*Paneluskalimantanicus* Ochi, Kon & Barclay, 2009	accepted	species
*Paragymnopleurusmaurus* (Sharp, 1875)	accepted	species
*Paragymnopleurusmaurusmaurus* (Sharp, 1875)	accepted	subspecies
*Paragymnopleurussparsus* (Sharp, 1875)	accepted	species
*Paragymnopleurussparsussparsus* (Sharp, 1875)	accepted	subspecies
*Paragymnopleurusspinotus* (Boucomont, 1914)	accepted	species
*Paragymnopleurusstriatus* (Sharp, 1875)	accepted	species
*Parascatonomusrudis* (Sharp, 1875)	accepted	species
*Parascatonomusanitidus* (Ochi & Kon, 2005)	accepted	species
*Parascatonomusaurifex* (Harold, 1877)	accepted	species
*Parascatonomusbrendelli* (Ochi, Kon & Barclay, 2008)	accepted	species
*Parascatonomusbundutuhanensis* (Ochi, Kon & Barclay, 2008)	accepted	species
*Parascatonomusdanumcupreus* (Krikken & Huijbregts, 2009)	accepted	species
*Parascatonomusdux* (Sharp, 1875)	accepted	species
*Parascatonomusgunsalami* (Ochi & Kon, 2005)	accepted	species
*Parascatonomuskatoi* (Ochi & Araya, 1996)	accepted	species
*Parascatonomuskatoiporingensis* (Ochi & Kon, 2005)	accepted	subspecies
*Parascatonomuskikutai* (Ochi & Kon, 2005)	accepted	species
*Parascatonomusliewi* (Ochi & Kon, 2005)	accepted	species
*Parascatonomusmonticupreus* (Krikken & Huijbregts, 2009)	accepted	species
*Parascatonomusporingensis* (Ochi & Kon, 2005)	accepted	species
*Parascatonomussarawacus* (Harold, 1877)	accepted	species
*Parascatonomussayapensis* (Ochi & Kon, 2005)	accepted	species
*Parascatonomussemiaureus* (Lansberge, 1883)	accepted	species
*Parascatonomussemicupreus* (Harold, 1877)	accepted	species
*Parascatonomustaichii* (Ochi, Kon & Barclay, 2008)	accepted	species
*Parascatonomustamijii* (Kon, Sakai & Ochi, 2000)	accepted	species
*Parascatonomusfujiokai* (Ochi & Araya, 1996)	accepted	species
*Parascatonomuspenicillatus* (Harold, 1879)	accepted	species
*Phacosomadytiscoides* Boucomont, 1914	synonym	species
*Phacosomagangkui* Ochi, Kon & Kikuta, 1997	synonym	species
*Phacosomamasumotoi* Ochi & Araya, 1996	synonym	species
*Phacosomaparantisae* Ochi, Kon & Kikuta, 1997	synonym	species
*Phacosomahikidai* Ochi, Kon & Kikuta, 1997	synonym	species
*Proagoderusschwaneri* (Snellen Van Vollenhoven, 1864)	accepted	species
*Proagoderuswatanabei* (Ochi & Kon, 2002)	accepted	species
*Sisyphusthoracicus* Sharp, 1875	accepted	species
*Synapsiscambeforti* Krikken, 1987	synonym	species
*Synapsiscambefortiporingensis* Ochi, Kon & Kawahara, 2008	synonym	subspecies
*Synapsisritsemae* Lansberge, 1874	accepted	species
*Yvescambefortiussarawacus* (Gillet, 1926)	accepted	species

**Table 3.** Description of variables within each dataset.

**Column heading**	**Description**
Dataset 1 (Core): Sampling Event and Occurrence Records of Dung Beetles (Coleoptera, Scarabaeinae) from Sabah, Malaysia	
parentEventID	An identifier for the broader event that groups this and other events. This is a globally unique identifier.
eventID	An identifier for the set of information associated with an event (something that occurs at a place and time). It is used as a unique identifier of each event and can be linked to available extensions of the core dataset. This is a globally unique identifier.
samplingProtocol	The names of, references to, or descriptions of the methods or protocols used during an event.
eventRemarks	Comments or notes about the event. Emphasis is placed on examining this column, as the data type and scale (i.e. trap-, site-, study-, taxonomic-) will determine the subsequent analysis that can be performed. This column also contains verbatim site name, transect name/no. and trap no. Cells containing more than one type of data are separated by ‘|’.
samplingEffort	The amount of effort expended during an event.
sampleSizeValue	A numeric value for a measurement of the size (time, duration, length, area or volume) or a sample in a sampling event.
sampleSizeUnit	The unit of measurement of the size (time, duration, length, area or volume) of a sample in a sampling event.
eventDate	The date-time or interval during which an event occurred. Examples: 2018-08-29 (some time during 29 August 2018); 1906-06 (some time in June 1906); 1971 (some time in 1971); 2007-03-01/2008-05-11 (some time during the interval between 1 March 2007 and 11 May 2008); 1900/1909 (some time during the interval between the beginning of the year 1900 and the end of the year 1909); 2007-11-13/15 (some time in the interval between 13 November 2007 and 15 November 2006).
country	Country in which event occurred.
countryCode	The standard code for the country in which the event occurred. Country code is as per ISO 3166-1-alpha-2 country code.
locality	The specific description of the place.
decimalLatitude	The geographic latitude (in decimal degrees, using the spatial reference system given in geodeticDatum) of the geographic centre of the event.
decimalLongitude	The geographic longitude (in decimal degrees, using the spatial reference system given in geodeticDatum) of the geographic centre of the event.
geodeticDatum	The ellipsoid, geodetic datum or spatial reference system (SRS) upon which the geographic coordinates given in decimalLatitude and decimalLongitude are based.
bibliographicCitation	A bibliographic reference of where the event was derived.
Dataset 2 (Extension): Occurrence	
parentEventID	An identifier for the broader event that groups this and other events. This is a globally unique identifier.
eventID	An identifier for the set of information associated with an event (something that occurs at a place and time). It is used as a unique identifier of each event and can be linked back to the core of this dataset. This is a globally unique identifier.
occurrenceID	An identifier for the occurrence. This is a globally unique identifier.
basisofRecord	The specific nature of the data record (i.e. MaterialCitation)
scientificName	The scientific name of the species, with full authorship and date information, if known.
organismQuantity	The number or value for the quantity or organism gathered from the event.
organismQuantityType	The type of quantification system used for the quantity of the organisms.
occurrenceStatus	A statement about the presence or absence of a taxon at a location.
taxonRank	The taxonomic rank of the most specific name in the scientificName.
type	The nature or genre of the resource (i.e. Event).
Dataset 3 (Extension): Reference	
eventID	A unique identifier for the reference that can be linked to the event in which it was derived from. This is a globally unique identifier.
bibliographicCitation	A bibliographic reference of the resource.
Dataset 4 (Core): Taxonomic Checklist of the Dung Beetles (Coleoptera, Scarabaeidae, Scarabaeinae) of Sabah, Malaysia	
taxonID	A unique identifier for the taxon and can be used to linked to available extensions of the core dataset. This is a globally unique identifier.
kingdom	The full scientific name of the kingdom in which the Taxon is classified.
phylum	The full scientific name of the phylum in which the Taxon is classified.
class	The full scientific name of the class in which the Taxon is classified.
order	The full scientific name of the order in which the Taxon is classified.
family	The full scientific name of the family in which the Taxon is classified.
subfamily	The full scientific name of the subfamily in which the Taxon is classified.
genus	The full scientific name of the genus in which the Taxon is classified.
subgenus	The full scientific name of the subgenus in which the Taxon is classified.
infragenericEpithet	The infrageneric part of a binomial name at ranks above species, but below genus.
specificEpithet	The name of the first or species epithet of the scientificName.
scientificName	The scientific name of the species, with full authorship and date information, if known.
scientificNameAuthorship	The latest known author of the scientific name.
nameAccordingTo	The reference to the source in which the specific taxon is defined. This is typically the latest authoritative taxonomic reference to species.
namePublishedIn	A reference for the publication in which the scientificName was originally established.
namePublishedInYear	The year in which the scientificName was published.
taxonomicStatus	The taxonomic status of the scientificName as determined by expert opinion.
acceptedNameUsage	The full name, with authorship and date information, if known, of the currently valid or accepted Taxon.
acceptedNameUsageID	An identifier of the name usage of the current valid or accepted taxon. This is a globally unique identifier.
originalNameUsage	The taxon name, with authorship and date information if known, as it originally appeared when first established. This is typically the basionym of the scientificName or senior/earlier homonym for replaced names.
originalNameUsageID	An identifier for the name usage in which the scientific name was originally established. This is a globally unique identifier.
taxonRank	The taxonomic rank of the most specific name in the scientificName.
taxonRemarks	Comments of notes about the taxon or name.
Dataset 5 (Extension): Reference	
taxonID	A unique identifier for the reference which corresponds to the bibliographic citation of the taxon occurrence. This is a globally unique identifier.
bibliographicCitation	A bibliographic reference of the resource.
